# Discriminating evidence – use and misuse of the drug-discrimination test in abuse potential assessment of novel CNS drugs

**DOI:** 10.1177/02698811251330780

**Published:** 2025-04-17

**Authors:** David J Heal, Sharon Lesley Smith, Jane Gosden, James Rowlett

**Affiliations:** 1 DevelRx Ltd, BioCity, Nottingham, UK; 2 Department of Life Sciences, University of Bath, Bath, UK; 3 Department of Psychiatry and Human Behavior, Center for Innovation and Discovery in Addictions, University of Mississippi Medical Center, Jackson, MS, USA

**Keywords:** Drug discrimination, rats, CNS drugs, methodology, predictive validity, translational validity

## Abstract

Nonclinical testing to predict the abuse potential of central nervous system (CNS) drug candidates is a mandatory part of the safety pharmacology assessment for medications seeking approval for human use. In the “standard model,” the drug candidate is tested to determine whether its psychoactive effects generalize to the discriminative cue of an abused drug that animals have been trained to recognize. However, CNS drugs with novel pharmacological mechanisms are challenging, and in response, the regulatory agencies have recommended alternative experimental designs. Variant 1: test the drug candidate in a series of drug-discrimination experiments that exemplify the major classes of abused drugs. Variant 2: use the drug candidate as a training cue. Back-test examples from established classes of abused drugs to see if they generalize to the drug candidate’s cue. We critically assessed the pharmacological and translational validity of these protocols. The standard model is underpinned by decades of research and refinement and has the highest degree of translational validity. Question marks exist over the validity of substitution results when the drug candidate has no affinity for known abuse-related targets. Published research does not support the use of either of the alternative models. On the contrary, these models have no pharmacological rationale and, consequently, no translational validity. The review contains a decision tree on the appropriate application of the standard drug-discrimination model, together with recommendations for adapting the test when characterizing the psychoactive properties of drug candidates acting on novel CNS targets.

## Glossary of terms

Primary pharmacology: Pharmacological action of the drug candidate that produces efficacy in the selected therapeutic indication.

Secondary pharmacology: Pharmacological actions of the drug candidate that produce effects unrelated to efficacy and could cause adverse events, reduce tolerability, or negatively impact safety.

On-target effect: Interaction between the drug candidate and molecular target(s), for example, receptor, ion channel, allosteric site, transporter, or enzyme, that mediates its therapeutic effect.

Off-target effects: Interaction between the drug candidate and molecular target(s) that are unrelated to its therapeutic effect and could cause adverse events, reduce tolerability, or negatively impact safety.

Positive control: The positive control used as the discriminative cue for training the animals must be a controlled drug, for example, a substance in schedules 1 to 5 (C-I to C-V) of the U.S. Controlled Substances Act, or C-1 to C-V of the U.K. Home Office Misuse of Drugs Act.

Negative control: Vehicle.

Reference comparator: A known drug that is included in the study to provide context and/or benchmark the results obtained with the drug candidate. The Reference comparator can either be a controlled drug or it can be a non-scheduled drug with an approved medical use in humans.

## Introduction

Investigating the human abuse potential of novel CNS-active drug candidates in animal models is not only a regulatory requirement (Committee for Medicinal Products for Human Use/European Medicines Agency, 2006; Center for Drug Evaluation and Research/Food and Drug Administration, 2017) but also an essential part of evaluating its safety profile for the benefit of prescribers, patients, and the public.

The abuse potential assessment is based on two nonclinical tests:

1. Drug discrimination investigates the drug candidate’s psychoactive properties to determine whether it produces CNS effects similar to those of a known substance of abuse.
2. Intravenous self-administration investigates whether the drug candidate’s psychoactive effects are sufficiently rewarding to initiate drug-seeking leading to psychological dependence.

The premise for these nonclinical testing procedures is that they can reliably predict whether the drug candidate is likely to have abuse potential in humans. Since these experiments are performed in animals, the accuracy of the prediction relies on the translational validity of the model. If the experimental design is sub-optimal or invalid, the model can generate positive or negative findings that do not translate to humans. This is a critically important issue in territories like Europe where regulatory agencies conduct their abuse risk assessments without requiring evidence from a human abuse liability trial in recreational drug users (Calderon et al., 2015). Preserving the translational validity of nonclinical models is particularly problematic when attempting to predict the abuse potential of CNS drug candidates with novel pharmacological mechanisms because no information on the risk of abuse in humans exists beyond the experience gained in Phase 1 and 2 clinical trials.

We have focused on the use of drug discrimination to evaluate CNS drug candidates with novel pharmacological mechanisms. The CHMP/EMA (2006) guidelines offer generic advice on the conduct and interpretation of findings from nonclinical drug discrimination tests. In contrast, the CDER/FDA (2017) guideline contains specific advice on the design, conduct, and interpretation of drug-discrimination studies, and in addition, scientists from the Controlled Substance Staff (CSS) of the Food and Drug Administration (FDA) provide advice and feedback on experimental protocols submitted to the agency for review.

Where there is a clear link between the pharmacology of the drug candidate and a known class of substance of abuse, the choice of positive control in the model is relatively straightforward. As an example, for a drug candidate that potentiates dopaminergic neurotransmission investigating whether it generalizes to the cue elicited by an indirect dopamine agonist such as d-amphetamine or cocaine is a pharmacologically and translationally valid choice. On the other hand, when the drug candidate has a novel CNS mechanism and does not interact with any target that produces the psychoactive effects of known substances of abuse, which substance of abuse to select as the discriminative cue for substitution testing with the drug candidate is a difficult decision.

In this review, we have critically assessed (i) the experimental variants of the drug discrimination advocated by drug regulators to characterize the psychoactive properties of novel CNS compounds, (ii) identified their strengths and weaknesses, and, critically, (iii) the translational validity of the results generated by each of these experimental models.

The protocol designs for nonclinical drug-discrimination studies described below have been advocated by the European Medicines Agency (EMA) (CHMP/EMA, 2006) and FDA (CDER/FDA, 2017) (CSS written advice to sponsors; data on file).

### Standard experimental design

1. Drug candidate is tested in animals trained to discriminate a selected abused substance from vehicle (CHMP/EMA, 2006; CDER/FDA, 2017).

This form of the drug-discrimination test has been developed for use in animals (Colpaert, 1999; Swedberg, 2016) and humans (Bolin et al., 2018; Lamb and Henningfield, 1994), and the translational validity of the data is well established (Horton et al., 2013; Kamien et al., 1993; Kelly et al., 2003).

### Alternative experimental designs

1. Drug candidate is used as the training cue. The animals are trained to discriminate between the drug candidate and vehicle. If the animals can reliably discriminate between the psychoactive effects of the drug candidate and vehicle, representative examples of substances of abuse from different pharmacological classes are tested to determine if they generalize to the stimulus elicited by the drug candidate (CSS written advice to sponsors; data on file).
2. Drug candidate is investigated in multiple studies with animals trained to recognize representatives from major classes of abused substances (e.g., a hallucinogen, a stimulant, a cannabinoid, a µ-opioid receptor agonist, sedative agent; CSS written advice to sponsors; data on file). This testing to be performed even when a drug candidate with a novel mechanism of action has been screened and found to have no affinity for the molecular targets that produce the psychoactive effects of known drugs of abuse. Drug candidate is tested in each study to see if it generalizes to one or more of the classes of abused drugs.

## Interpreting results from drug-discrimination experiments

The CHMP/EMA (2006) guidelines offer no advice on the interpretation of results from drug-discrimination experiments. It does add the caveat that “*However, generalization with an active substance known to cause dependence in itself is not necessarily indicative of dependence potential. . .*” (N.B. CHMP/EMA uses “dependence” as a term to describe a drug’s potential for abuse without necessarily implying that the drug can cause physical dependence.)

The CDER/FDA (2017) guidelines recommend that “Full generalization” should be defined as occurring when the animal makes >80% presses on the lever assigned to the training drug, while “No generalization” is when the animal lever-presses <20% lever paired with the training drug, that is, >80% responding on the vehicle-paired lever. Partial generalization applies to 21%–79% responding on the drug-paired lever. Interpreting the result for a drug candidate that produces responses in this “grey zone” poses the question what level of partial generalization is a cause for concern. CDER/FDA (2017) advises “*For regulatory purposes, partial generalization between 60% and 80% suggests that the test drug produces an interoceptive cue that has some similarity to the training drug”* indicating that this outcome should be treated as a yellow rather than a red flag for abuse potential.

## Scientific rationale and pharmacological foundations of the drug-discrimination test

### Pharmacological specificity of the interoceptive cue

The scientific rationale for evaluating CNS compounds is based on the premise that humans abuse prescription drugs and other CNS-active substances because the psychoactive effects they generate give a pleasurable experience. It follows that if a different CNS compound, for example, a CNS drug candidate, elicits psychoactive effects that are identical or very similar to those generated by a known substance of abuse, the prediction is it will probably evoke a similar pleasurable experience in humans that constitutes an abuse risk.

The role of the drug-discrimination test is to detect abuse potential in novel drug candidates. For this purpose, the key assumption that has been adopted by the regulatory agencies’ is the pharmacological mechanism that produces the discriminative cue for the substance of abuse that the animals are trained to recognize is identical to the one responsible for its human abuse liability. From this assumption, it follows that generalization accurately predicts that the psychoactive effects produced by the drug candidate are indistinguishable from the abused substance, and therefore, the drug candidate shares its potential for human abuse. As we will illustrate, the results derived from drug-discrimination testing are not only pharmacologically specific but also more nuanced and informative than the simple “yes/no” interpretation implied in the key assumption.

In support of the model’s validity, the discriminative cue produced by a psychoactive drug is invariably CNS-mediated. As an example, when lysergic acid diethylamide (LSD) is used as a training cue, 5-HT_2A_ receptor agonists that do not cross the blood-brain barrier fail to generalize to its cue, but generalization to LSD can be elicited by central injection of LSD and other 5-HT_2A_ receptor agonists (Gresch et al., 2005; White and Appel, 1982).

To compare the translational validity of the data generated by the alternative protocol designs for drug discrimination, we used experimental data generated in our research together with published findings from a comprehensive search of the literature including our recent review on assessing the abuse potential of biological and synthetic cannabinoids (Heal et al., 2024a). Searches using the terms “drug discrimination,” “THC,” “benzodiazepines,” “GABA,” “midazolam,” or “lorazepam” were performed. Wherever possible, we included all species used in drug-discrimination experiments and the relevant data from every article is presented in Tables 2–4. The only omissions were when other publications appeared reporting the same finding(s).

Table 1 provides an overview of published findings from drug-discrimination experiments performed with archetypical compounds from all of the major pharmacological classes of abused drugs. In each pharmacological class, the interoceptive cue produced by the substance of abuse used to train the animals was mimicked by compounds having an identical or very similar pharmacological mechanism of action, and in addition, the pharmacological specificity of each cue was confirmed by blockade with selective antagonists (Table 1). To provide greater granularity on this topic, we selected the cannabinoid, (−)Δ^9^-tetrahydrocannabinol [(−)Δ^9^-THC] as an example to demonstrate the pharmacological specificity of its discriminative cue. The results shown in Table 2 unequivocally demonstrate that (−)Δ^9^-THC’s discriminative cue is blocked by the selective CB_1_ antagonist rimonabant, and furthermore, rimonabant similarly prevents generalization to (−)Δ^9^-THC by a range of synthetic cannabinoid CB_1_/CB_2_ agonists. The discriminative cue produced by (−)Δ^9^-THC is not influenced by the selective CB_2_ antagonist, SR-144528, or by antagonists of dopamine, serotonin, norepinephrine, opioid, or cholinergic receptors.

**Table 1.** Pharmacological specificity of the discriminative cues elicited by various substances of abuse routinely employed in drug-discrimination testing for abuse potential assessment.

Species	Discriminative cue	Cross-substituting compound	Antagonist blocking generalization	Ref
Rat	d-Amphetamine	Lisdexamfetamine	Olanzapine (D_2_ + 5HT_2A_ antagonist)	Hutson et al. (2016)
Rat	d-Amphetamine	Methamphetamine (DA/NA releaser), bupropion (DAT inhibitor)	N/D	Heal et al. (1992)
Rat	d-Amphetamine	N/D	Haloperidol (D_2_ antagonist), inactive phenoxybenzamine (α antagonist), inactive propranolol (β antagonist)	Schechter and Cook (1975)
Rat	d-Amphetamine	Apomorphine (non-selective D_1–5_ agonist), piribedil (D_2_/D_3_ agonist)	N/D	Schechter (1977)
Rat	d-Amphetamine	Quinpirole (D_3_ agonist)	N/D	Arnt and Hyttel (1990)
Rat	d-Amphetamine	Quinpirole (D_3_ agonist)	Haloperidol (D_2_ antagonist), SCH23390 (D_1_ antagonist)	Callahan et al. (1991)
Mouse	Cocaine	Quinpirole (D_3_ agonist), pramipexole (D_3_ agonist)	N/D	Collins et al. (2014)
Rat	DOM	DOI (5-HT_2A/2C_ agonist), LSD (5-HT_2A/2C_ partial agonist), mescaline (5-HT_2A/2C_ partial agonist)	N/D	Halberstadt et al. (2020)
Rat	DOI	LSD (5-HT_2A/2C_ partial agonist)	Ketanserin (5-HT_2A/2C_ antagonist), spiperone (5-HT_2A_ + D_2_ antagonist)	Chojnacka-Wojcik and Klodzinska (1997)
Rat	DOI	N/D	MDL 100,907 (5-HT_2A_ antagonist)	Schreiber et al. (1994)
Monkey	Midazolam	Flunitrazepam (GABA-A PAM), allopregnanolone (GABA-A PAM)	Flumazenil (benzodiazepine antagonist)	Gerak and France (2012)
Monkey	Morphine	Fentanyl (µ agonist), oxymorphone (µ agonist), methadone (µ partial agonist)	N/D	Schaefer and Holzman (1977)
Rat	Morphine	Buprenorphine (µ partial agonist)	N/D	Shannon et al. (1984)
Monkey	Heroin	N/D	Naltrexone (µ antagonist), 3-methoxynaltrexone (µ antagonist)	Bowen et al. (2002)
Monkey	Morphine	N/D	Naltrexone (µ antagonist), 3-methoxynaltrexone (µ antagonist)	Bowen et al. (2002)
Rat	Phencyclidine	Ketamine (NMDA antagonist)	N/D	Heal et al. (2018a)
Rat	Phencyclidine	Methoxetamine (NMDA antagonist), eticyclidine (NMDA antagonist), tenocyclidine (NMDA antagonist)	N/D	Berquist et al. (2018)

DA: dopamine; NA: noradrenaline; N/D: not determined; IA: inactive; LSD: lysergic acid diethylamide; DOI: 2,5-dimethoxy-4-iodoamphetamine; DOM: 2,5-dimethoxy-4-methylamphetamine; BDZ: benzodiazepine.

**Table 2.** Pharmacological specificity of the (−)Δ^9^-tetrahydrocannabinol interoceptive cue demonstrated by antagonist blockade.

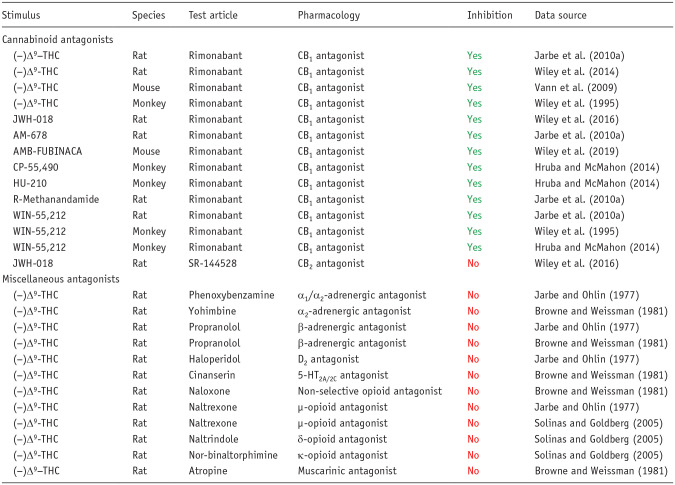

Interpretation of the results: Yes: antagonist prevented full generalization (> 80% responding on the drug-assigned lever) or caused a rightward shift in the dose–response curve for generalization to the (−)Δ^9^-THC cue; No: antagonist did not prevent generalization to (−)Δ^9^-THC cue.

The findings presented in Table 3 show only compounds that are CB_1_ partial or full agonists substituted for (−)Δ^9^-THC in drug-discrimination testing, and this finding has been replicated across all species used for these experiments. In a more comprehensive analysis of cannabinoid compounds in (−)Δ^9^-THC-cued drug-discrimination experiments, we discovered that more than 35 botanical and synthetic psychoactive CB_1_ agonists have been tested, and all have been shown to generalize to (−)Δ^9^-THC (Heal et al., 2024a). In contrast, non-psychoactive cannabinoids that lack CB_1_ agonist activity do not substitute for (−)Δ^9^-THC (Table 3; Heal et al., 2024a). Many CNS-active compounds have been profiled in (−)Δ^9^-THC-cued drug discrimination, including several types of substances of abuse, for example, serotonergic psychedelics, entactogens, catecholaminergic stimulants, benzodiazepines, barbiturates, opioid agonists, nicotine, and ethanol. As reported in Table 3, CB_1_ receptor partial and full agonists were the only compounds that substitute for (−)Δ^9^-THC. Drugs evoking CNS effects similar to (−)Δ^9^-THC, for example, sedation, euphoria, perceptual and sensory changes, and/or intoxication, through different pharmacological mechanisms did not generalize to the (−)Δ^9^-THC discriminative cue (Table 3).

**Table 3.** Pharmacological specificity of the interoceptive cue in drug discrimination exemplified by generalization to (−)Δ^9^-tetrahydrocannabinol.

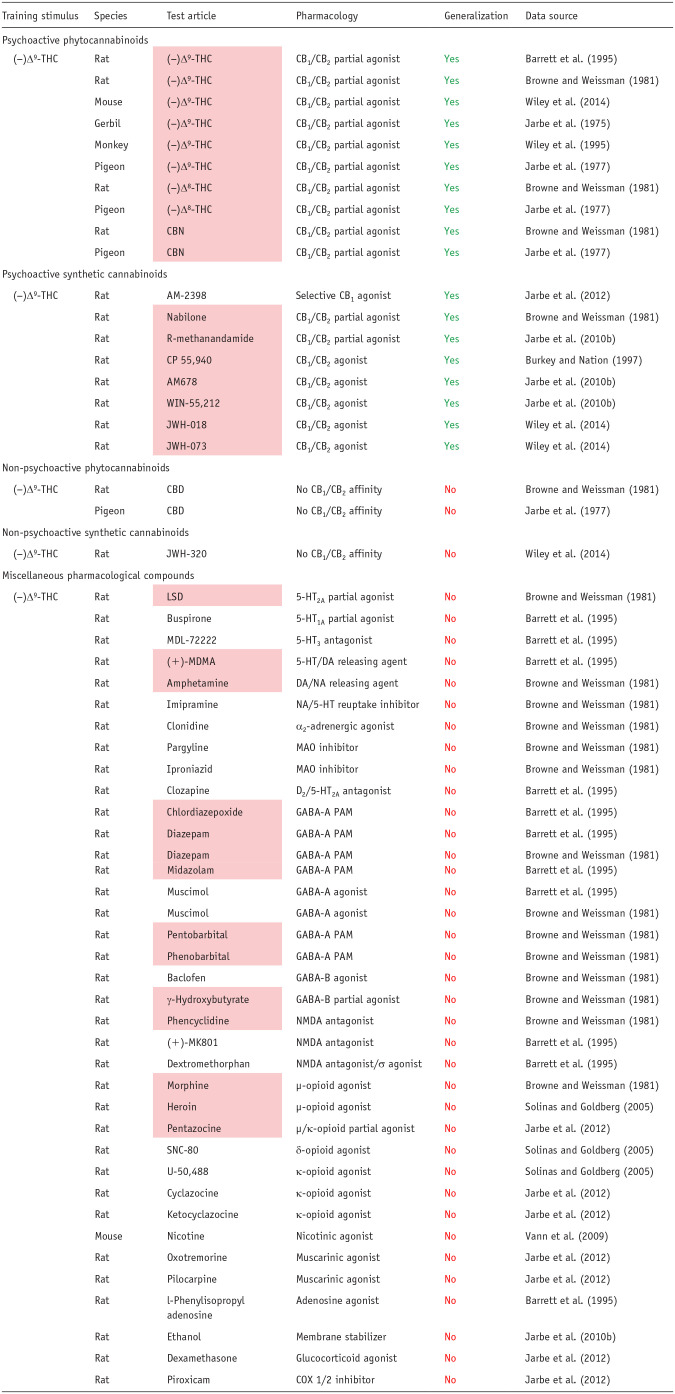

Interpretation of the results: Yes: test article achieved full generalization to the (−)Δ^9^-THC cue (⩾80% responding on the drug-assigned lever); No: test article produced either no generalization or partial generalization to (−)Δ^9^-THC (⩽20% and 21%–79% responding on the drug-assigned lever, respectively). Compounds in the pink shaded cells are scheduled under the U.S. Controlled Substances Act.

(−)Δ^9^-THC: (−)Δ^9^-tetrahydrocannabinol; CBD: cannabidiol; CBN: cannabinol; (±)MDMA: (±)3,4-methylenedioxy-methamphetamine; LSD: lysergic acid diethylamide.

To further illustrate the pharmacological specificity of discriminative stimulus, we have provided an overview of the drug-discrimination literature on benzodiazepines. The benzodiazepines and related drugs act as positive allosteric modulators (PAMs) of the γ-aminobutyric acid type A (GABA_A_) receptor by interacting with a binding site distal to the GABA site and enhancing GABA-induced chloride conductance (Sigel and Ernst, 2018; Sieghart and Savic, 2018). Benzodiazepines representing the classical anxiolytics and sedatives readily serve as discriminative stimuli, including diazepam, alprazolam, and zolpidem, which is not structurally a benzodiazepine but shares a similar mechanism of action (Table 4). Antagonists selective for the benzodiazepine site on the GABA_A_ receptor consistently attenuate the discriminative stimulus effects of benzodiazepines and shift the benzodiazepine dose–response function for discriminative effects rightward in a surmountable fashion (Table 4). Multiple subtypes of the GABA_A_ receptor exist, and antagonists that show selectivity for the subtype containing α1 subunits (α1GABA_A_ subtypes) antagonize the discriminative cue of benzodiazepine-type drugs (Table 4). By contrast, antagonists specific for the primary GABA binding site or the picrotoxin binding site on the GABA_A_ receptor do not alter the discriminative effects of benzodiazepines. Relatively few reports of tests for antagonism with ligands specific for non-GABA_A_ receptors exist, but almost all of these antagonists were ineffective at blocking the benzodiazepine cue. The only exception was the unreplicated blockade of diazepam’s cue by the α_2_-adrenoceptor antagonist, yohimbine (Table 4). Overall, these results indicate that the discriminative cue associated with essentially all benzodiazepines evaluated to date reflect action at the benzodiazepine-sensitive site on the GABA_A_ receptor.

**Table 4.** Pharmacological specificity of the benzodiazepine interoceptive cue demonstrated by antagonist blockade.

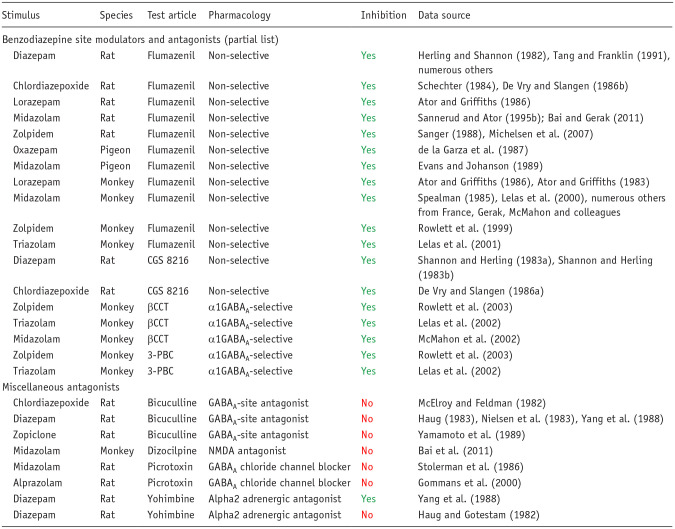

Interpretation of the results: Yes: antagonist prevented full generalization (⩾80% responding on the drug-assigned lever) or caused a rightward shift in the dose–response curve for generalization to the benzodiazepine cue; No: antagonist did not prevent generalization to benzodiazepine cue.

NMDA = N-methyl-D-aspartate

A considerable body of literature exists, beginning in the early 1980s, regarding the ability of benzodiazepines to generalize to different drugs and compounds with varying pharmacological mechanisms of action. To be concise, we focused on two representative training drugs, midazolam and lorazepam, for which the most data are available. Moreover, as shown in Table 5, the midazolam and lorazepam cues differ in pharmacological specificity, although generalization to a broad range of benzodiazepine-type drugs is evident for both drugs (Table 5). Note that this observation of complete cross-generalization among benzodiazepines is consistent across all classical benzodiazepines (e.g., diazepam, triazolam; (Lelas et al., 2001; Shannon and Herling, 1983b). Other GABA_A_ PAMs show less consistent effects, with barbiturates fully substituting in some studies using midazolam-trained subjects but not in others, whereas lorazepam-trained rats and monkeys have consistently shown no generalization to a variety of barbiturates. These results have led to the conclusion that the lorazepam cue has a greater degree of pharmacological specificity than other benzodiazepine cues (Ator and Griffiths, 1986, 1989, 1997). Similarly, neuroactive steroids that act as GABA_A_ PAMs fully substituted for midazolam in rats and monkeys but did not substitute in lorazepam-trained rats (note that the neuroactive steroid did not substitute in triazolam-trained monkeys, but did so in triazolam-trained rats; the reasons for these discrepancies are unknown but may involve species, route of administration, and training dose used (Gunter et al., 2016; Lelas et al., 2002). Regardless of experimental conditions, ligands that either act directly on the GABA site of the GABA_A_ receptor, or facilitate GABA transmission, lack benzodiazepine discriminative effects (see also de la Garza et al., 1987; Gommans et al., 2000; Lelas et al., 2001; Shannon and Herling, 1983b; Yamamoto et al., 1989). Collectively, these findings suggest that the highest probability of substitution across benzodiazepine training conditions is with another benzodiazepine site ligand. Enhancing GABA’ergic signaling by a mechanism that involves allosteric modulation at a different GABA_A_ site or GABA action itself substantially decreases the likelihood of substitution.

**Table 5.** Pharmacological specificity of the interoceptive cue in drug discrimination exemplified by generalization to benzodiazepines, focused on midazolam and lorazepam as training stimuli.

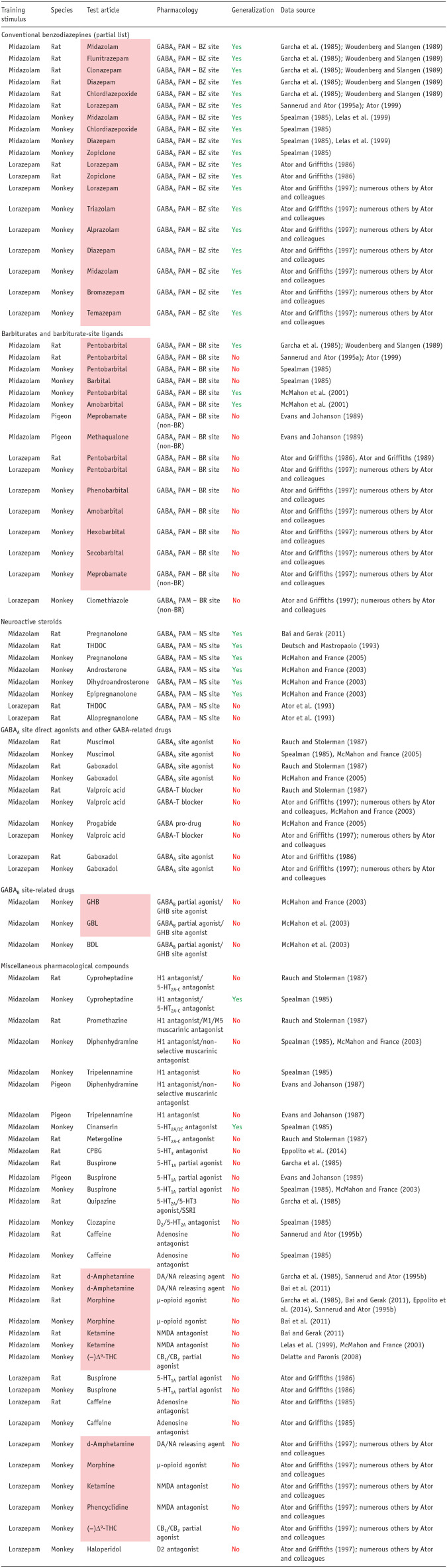

Interpretation of the results: Yes: test article achieved full generalization to the benzodiazepine cue (⩾80% responding on the drug-assigned lever); No: test article produced either no generalization or partial generalization to the benzodiazepine (⩽20% and 21%–79% responding on the drug-assigned lever, respectively). Compounds in the pink shaded cells are scheduled under the U.S. Controlled Substances Act.

PAM: positive allosteric modulator; BZ: benzodiazepine; BR: barbiturate; non-BR: chemical structure distinct from barbiturates; NS: neuroactive steroid; GHB: gamma-hydroxybutyrate; (−)Δ^9^-THC: (−)Δ^9^-tetrahydrocannabinol; CPBG: 1-(m-chlorophenyl)-biguanide; THDOC: 5α-pregnane-3α,21-diol-20-one.

When drugs with non-GABA_A_ mechanisms of action have been evaluated for substitution in benzodiazepine-trained subjects, the pharmacological specificity of this cue becomes strikingly evident (Table 5). The only reported exception has been with ligands with 5-HT receptor antagonist properties. It should be noted that this finding was not consistent across studies and species and, using the same methodologies, was not observed with different training drugs (i.e., zolpidem, (Rowlett et al., 1999); triazolam, (Lelas et al., 2002)). A key consideration for these findings is that many of these drugs share general behavioral effects with benzodiazepines, such as anxiolysis (e.g., buspirone) and sedative-motor effects (e.g., diphenhydramine, ketamine, morphine, and haloperidol), yet do not elicit benzodiazepine-like discriminative stimulus effects. Therefore, for more general drug effects, such as sedation, the benzodiazepine cue has low predictive validity, and we recommend that it should not be used in this context. With respect to predicting abuse liability, the benzodiazepine cue will reliability predict drug candidates that act at the benzodiazepine site on the GABA_A_ receptor only, with false negatives a high possibility even for other GABA_A_ PAMs. Therefore, drug-discrimination analysis for abuse liability should be carefully considered in this context. Finally, substantial research points to training situations involving relatively high doses that resulted in *increased* pharmacological specificity (e.g., Rowlett et al., 2003; Sannerud and Ator, 1995b), raising critical questions about the utility of this procedure for identifying abuse liability (or other associated behavioral effects) under these experimental conditions.

Although many drug candidates are highly selective for a specific target, others produce their therapeutic effect through multiple pharmacological mechanisms. Some CNS compounds have off-target interactions that cause side effects and limit the therapeutic dose range. Therefore, the possibility exists of these off-target actions posing an abuse risk when the drug is taken in quantities that exceed the recommended therapeutic dose range. In turn, it raises the question whether the potential abuse risk posed by a drug candidate’s secondary therapeutic mechanism or off-target actions can be detected by drug discrimination. To address this question, we compared the results observed with various monoaminergic drugs in d-amphetamine-cued drug discrimination with their magnitude of effect on extracellular dopamine in the striatum and/or nucleus accumbens measured by in vivo microdialysis (Table 6). Only drugs that produced substantial increases in dopamine signaling in these brain regions yielded high-level partial or full substitution for d-amphetamine. Moreover, although reuptake inhibitors of noradrenaline (NARIs) or serotonin + noradrenaline (SNRIs) markedly increased synaptic dopamine levels in the prefrontal cortex (PFC) as a consequence of blocking noradrenaline reuptake transporters (Bymaster et al., 2002; Higashino et al., 2014; Nirogi et al., 2013; Umehara et al., 2013), they do not substitute for d-amphetamine in drug discrimination. Therefore, not only is a substantial increase in dopaminergic signaling a prerequisite for substitution to d-amphetamine, the increase must also be in specific brain regions for generalization to occur.

**Table 6.** Profiles of various monoaminergic drugs in the d-amphetamine-cued drug-discrimination test.

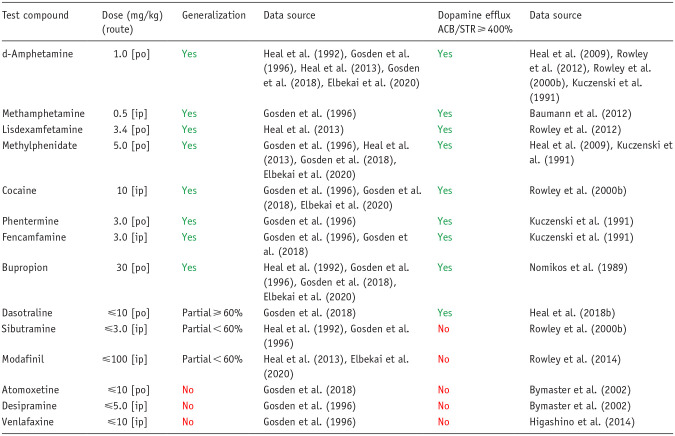

ACB: nucleus accumbens; STR: striatum.

To be clear, the lack of cross-substitution observed with these compounds is a translationally valid result. Using sibutramine as an example, its therapeutic effects as an anti-obesity drug were mediated by combined inhibition of noradrenaline and serotonin reuptake (Heal et al., 1998) and dopamine reuptake inhibition was an off-target pharmacological effect (Heal et al., 1998; Rowley et al., 2000a). The moderate increase in dopamine signaling produced by sibutramine failed to produce generalization to d-amphetamine (Table 6), which was the correct outcome because sibutramine also failed to produce d-amphetamine-like stimulant effects in drug-experienced human volunteers (Schuh et al., 2000).

Together, the findings support the following conclusions:

1. The interoceptive cue elicited by the training drug is pharmacologically specific.
2. Evidence for the specificity of the interoceptive cue is provided by experiments using selective antagonists to block the cue, and substitution studies with compounds from a range of pharmacological classes.
3. Drug candidates exhibiting a similar pharmacological profile to the training drug, for example, eliciting euphoriant, intoxicating, perceptual, sensory, stimulant, sedative, proconvulsant, anesthetic, or hallucinogenic effects, will not substitute in drug-discrimination.
4. The test article must express the specific pharmacological characteristics of the interoceptive cue for substitution to occur.
5. When drug discrimination is employed to detect the interoceptive cue elicited by a drug candidate’s off-target actions, for example, the dopaminergic actions of sibutramine, the probability of observing substitution is extremely low. This result as exemplified by sibutramine is likely to be translationally valid.

### The pharmacological link between the interoceptive cue and abuse potential

The premise that the pharmacological mechanism responsible for producing the interoceptive cue elicited by a substance of abuse, its potential to serve as a positive reinforcer or hallucinogen, and, ultimately, its liability for abuse is strongly supported by observations using selective antagonists. Examples are the psychoactive cannabinoids where it has been shown that the selective CB1 antagonist, rimonabant, prevented the psychoactive effects of smoked cannabis and decreased scores on the Addiction Research Center Inventory “marijuana” scale in experienced cannabis users (Huestis et al., 2007). This observation is consistent with reproducible evidence showing that the discriminative cue produced by (−)Δ^9^-THC and other naturally occurring and synthetic CB_1_/CB_2_ agonists is blocked by rimonabant (Table 2). The 5-HT_2A_/5-HT_2C_ antagonist, ketanserin, blocks the panoply of psychotomimetic, sensory, perceptual, and somatic effects that encompass the psychedelic experience in humans evoked by LSD (Becker et al., 2023; Holze et al., 2021; Preller et al., 2017) or dimethyltryptamine (DMT) (Valle et al., 2016). These findings accord with the discriminative cue produced by psychedelics like LSD, 2,5-dimethoxy-4-iodoamphetamine (DOI), and DMT being prevented by ketanserin and the 5-HT_2A_-selective antagonists, ritanserin, and MDL-100,907 (Carbonaro et al., 2015; Colpaert et al., 1985; Meert et al., 1989; Schreiber et al., 1994; Smith et al., 2003) (Table 1). The excellent correlation that exists between the potency of compounds to substitute for DOM in drug discrimination and to produce psychedelic effects in humans (Glennon et al., 1982; Halberstadt et al., 2020) provides further evidence in support of this hypothesis. For benzodiazepines, the benzodiazepine binding site on the GABA_A_ receptor that mediates the benzodiazepine cue (Tables 4 and 5) also is the site of action of addictive effects for this drug class (Engin, 2023). As a potent releaser of dopamine and noradrenaline, d-amphetamine has a dual mechanism of action that could result in the generation of a complex discriminative cue. Blockade of the d-amphetamine’s cue by dopaminergic antagonists, but not adrenergic antagonists (Table 1) demonstrate that the cue is exclusively dopamine mediated, a result that is supported by microdialysis data (Table 6). Consistent with this hypothesis, dopamine D_2_ antagonists, risperidone and aripiprazole, blocked the discriminative cue and reinforcing effects of d-amphetamine in human volunteers (Grob et al., 2011; Lile et al., 2005).

The link is not absolute because there are instances where a drug candidate will substitute for a substance of abuse, but post-approval experience has taught that the former poses no abuse risk in the “real world.” Various dopamine receptor full and partial agonists have been shown to generalize to d-amphetamine (see e.g., Table 1), and in addition, they also serve as positive reinforcers in self-administration (Freeman et al., 2012; Manzardo et al., 2001; Woolverton et al., 1984). When reviewing these “false positives,” it is important to consider the potential contribution of differences in the physiology of humans and these other species. Rats do not have an emetic reflex, and monkeys are highly resistant to the emetic effects of the dopamine agonists; hence, these species are better suited to access the rewarding effects of dopamine agonists without experiencing their aversive pro-emetic actions. Not all “false positive” hits in drug discrimination can be so easily explained; for example, the selective serotonin reuptake inhibitors (SSRIs), fluoxetine and clomipramine, have been reported to generalize to the mixed serotonergic/dopaminergic discriminative cue produced by 3,4-methylenedioxy-methamphetamine (MDMA; Webster et al., 2017).

To summarize, the evidence demonstrates that although the pharmacology of the interoceptive cue elicited by a substance of abuse is identical to the one mediating its rewarding/reinforcing effects in most cases, there are exceptions, and therefore, substitution for a substance of abuse is not a definitive signal of an abuse risk. For this reason, “hits” in drug-discrimination experiments should be interpreted with caution. This stance has been adopted in the CHMP/EMA (2006) guidelines. A similarly cautious interpretation is stated in the CDER/FDA (2017) guideline “*If the test drug produces full or partial generalization to the training drug (a known drug of abuse), it may have abuse potential.*”

## The challenge posed by drug candidates with novel pharmacological mechanisms

The pharmacological specificity of the drug-discrimination test underpins its value as a model for investigating whether novel drug candidates produce psychoactive effects similar to substances of abuse within the same pharmacological class. However, the model’s specificity is its weakness when attempting to evaluate the abuse potential of drug candidates with novel pharmacological mechanisms. As reported by ourselves and others, drug discrimination is a viable test when dealing with novel dopaminergic stimulants (Heal et al., 2014), psychedelics and entactogens (Heal et al., 2018a), NMDA antagonists (Heal et al., 2018a), κ-opioid receptor agonists (Heal et al., 2018a), psychoactive cannabinoids (Heal et al., 2024a), µ-opioid receptor agonists (Butelman and Kreek, 2018; Woods et al., 1988), and GABA_A_ PAMs (Lelas et al., 2000) (see also reviews by (Colpaert, 1999; Porter et al., 2018; Swedberg, 2016). However, the standard procedure is only appropriate if the drug candidate has a pharmacological mechanism of action that is identical or closely related to the substances of abuse to be used as the positive control (training cue) in the test.

This point is acknowledged in CDER/FDA (2017) guideline, which states “*Drug discrimination is dependent on mechanism of action, so only those test drugs that have pharmacological activity similar to that of the training drug will likely produce a significant degree of generalization to the training drug. Thus, if the test drug has a novel mechanism of action, sponsors may prefer to propose an alternative approach for identifying an appropriate training drug.*”

**Figure 1.**
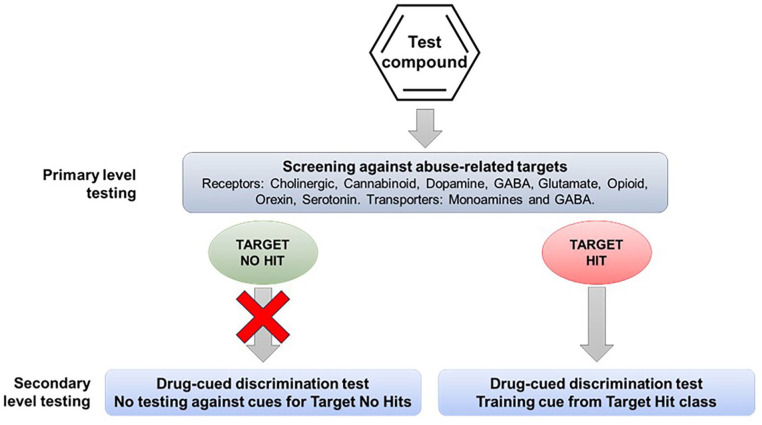
Drug-discrimination testing based on the pharmacological characteristics of the drug candidate. The drug candidate is pharmacologically characterized to define its proposed therapeutic mode of action and possible off-target interactions with targets mediating the actions of known substances of abuse. Drug-discrimination testing is performed using a representative substance of abuse as the training cue from the pharmacological class that is a “hit” for the drug candidate.

One of the first steps in the abuse potential assessment is in vitro screening to determine whether the drug candidate has affinity for any of the targets that mediate the actions of known substances of abuse, for example, receptors, allosteric sites, ion channels, and transporters. If the drug candidate, even one with a novel mechanism of action, interacts with one or more of these abuse-related targets, the information can be used to select appropriate positive controls for drug-discrimination testing (see Figure 1). However, pharmaceutical companies are increasingly developing drug candidates with novel pharmacological mechanisms that are devoid of off-target affinity for known abuse-related targets. This group of CNS drug candidates poses the greatest challenge when trying to design translationally valid drug-discrimination experiments.

Most abuse potential assessments are performed after the drug candidate has demonstrated efficacy in Phase 2 “proof of concept” trials at which point the therapeutic indication, clinically effective dose range, and drug exposure levels in patients will be known. In addition, the behavioral effects of the compound will have been investigated in CNS Safety Pharmacology (Irwin profile and/or functional observation battery (FOB)) and in acute and short-term toxicity testing in two species. The profile of adverse events produced by the drug candidate in normal, healthy volunteers and patients also helps to create a picture of the compound’s psychoactive properties that can be used collectively to select a controlled substance with a similar profile to use as the positive control in drug discrimination. The use of this approach is described and endorsed in the FDA’s guidance document (CDER/FDA, 2017).

### Drug-discrimination testing using a substance of abuse as the training cue

If the therapeutic mode of action of the drug candidate is shared, for example, novel CB1 receptor agonist, or similar, for example, monoacylglycerol lipase inhibitor or fatty acid amide hydrolysis inhibitor to enhance endocannabinoid signaling, there is a scientific rationale for evaluating the drug candidate in a conventional drug-discrimination experiment (Figure 1). A scientific argument can also be made for using this protocol if the drug candidate has off-target affinity for an abuse-related target. The evidence presented in our analysis shows drug-discrimination testing can reliably predict the psychoactive effects that the drug candidate is likely to produce in humans, and probably also, its potential to serve as a positive reinforcer. The main weakness is that the pharmacological specificity of the drug-discrimination test can generate false positive and false negative results for drug candidates, for example, dopamine agonists substituting for d-amphetamine and GABA_A_ PAMs failing to substitute for lorazepam.

When the therapeutic mechanism of the drug candidate is not linked with the pharmacology of known substances of abuse and screening for off-target effects yields no “hits,” there is no clear rationale for the choice of a substance of abuse to employ as the positive control for the drug-discrimination test. The recommended option (CDER/FDA, 2017) is to select the positive control choice based on a combination of the drug candidate’s intended therapeutic indication, its neuropharmacological effects in animals, and adverse events in humans. Experience using this approach has shown that without a shared link between the pharmacology of the drug candidate and the abused drug used to train the rats, substitution or ⩾60% partial substitution does not occur, for example, sibutramine and d-amphetamine (Table 6), cannabidiol and midazolam (Gray et al., 2022), ulotaront and d-amphetamine or MDMA (Synan et al., 2022), and soticlestat and ketamine (Heal et al., 2024b).

### Exploring the psychoactive properties of a drug candidate by drug-discrimination testing in multiple studies using substances of abuse from different pharmacological classes

**Figure 2.**
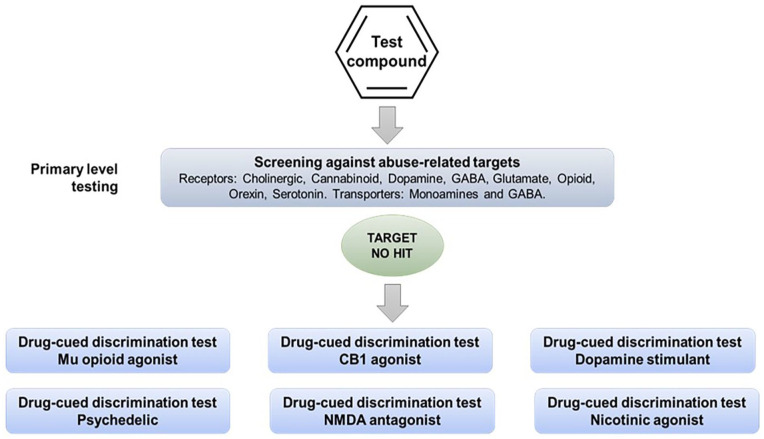
Alternative experimental design 1. Multiple drug-discrimination tests recommended when the therapeutic mechanism of the drug candidate is not linked with the pharmacology of known substances of abuse and screening for off-target effects yields no “hits.”

When the therapeutic mechanism of the drug candidate is not linked with the pharmacology of a known substance of abuse, and screening for off-target effects yields no “hits,” an alternative testing approach is to screen its psychoactive properties in a series of drug-discrimination studies using representatives from the major pharmacological classes of abused substances (Figure 2). This approach was first proposed by (Huskinson et al., 2014) who suggested that the psychotropic characteristics of a drug candidate with a novel pharmacological mechanism of action should be investigated in multiple substitution tests with a separate group of animals trained to discriminate a prototypical drug from the different classes of substances of abuse, for example, a hallucinogen, cannabinoid, stimulant, opioid, benzodiazepine, NMDA antagonist, entactogen, and kappa agonist. The FDA has accepted this approach and has recommended it to sponsors seeking advice on the nonclinical abuse assessment of CNS drug candidates (Confidential information held on file).

The ability to learn whether a drug candidate produces psychoactive effects that are similar to a single or multiple types of abused substances would be exceptionally informative in the abuse/dependence assessment because it could offer insights into the compound’s relative risk of abuse, and aid in devising the most relevant design for a study in drug-experienced human volunteers. Although the concept is superficially attractive, consideration of the pharmacological principles on which the model is based, or a cursory glance at reports of compound substitution in drug-discrimination experiments in publications would immediately flag that this experimental design will never deliver translationally predictive results.

As shown by the results for (−)Δ^9^-THC example in Table 2, the discriminative cue is not only specifically mediated by CB1 receptor activation, but the (−)Δ^9^-THC discriminative cue is also impervious to inhibition by antagonists of other neurotransmitters. Conversely, drugs that do not activate CB1 receptors do not substitute for (−)Δ^9^-THC (Table 3); this outcome occurred even when there is some similarity between their psychopharmacological effects and those of the psychoactive cannabinoids, for example, induce sedation, relaxation, euphoria, intoxication, dissociation, and/or psychomotor impairment. A similar overall pattern of results based on receptor mechanisms and not neuropharmacological effects was observed with the benzodiazepines (Tables 4 and 5).

As described in the previous section, even when the substance of abuse used to train the animals has been carefully selected based on similarity between to the psychoactive profile of novel CNS drug candidate, the chance of observing full or ⩾60% substitution is remote. Stated another way, even when there is shared pharmacology between drug candidate and the substance of abuse used as the positive control, unless there is substantial overlap, there is a very low probability of observing full or ⩾60% substitution (Table 6).

The role of in vitro screening against a comprehensive panel of abuse-related targets is a recommended preliminary step in determining whether a drug candidate poses an abuse risk (CHMP/EMA, 2006; CDER/FDA, 2017). What our analysis has revealed is unless there is a pharmacological interaction between the drug candidate and the target that produces the psychoactive effect of an abused drug class, for example, µ-opioid, CB_1_, 5HT_2A_ or NMDA receptors, GABA_A_ allosteric site, and dopamine or serotonin reuptake transporters, there is no scientific justification to conduct drug-discrimination testing against that specific cue. Moreover, in the remote likelihood that substitution did occur, the result will be a misleading abuse potential signal if the effect is not reversed by a selective antagonist (Figure 4).

Finally, there is no scientific rationale to support advice from regulatory agencies suggesting that sponsors should conduct discrimination testing of a drug candidate against cues associated with a dopaminergic stimulant, a psychedelic, a cannabinoid, benzodiazepine, a µ-opioid agonist, an NMDA antagonist if it has no pharmacologically relevant affinity for the molecular targets that produce the stimulus cue. According to the evidence presented, this approach has a negligible chance of observing cross-substitution. Overall, this approach necessitates an ethically debatable use of animals, and it is both time- and resource-intensive making the cost/benefit relationship untenable in virtually any conceivable circumstance.

### Drug-discrimination testing using the drug candidate as the discriminative cue

**Figure 3.**
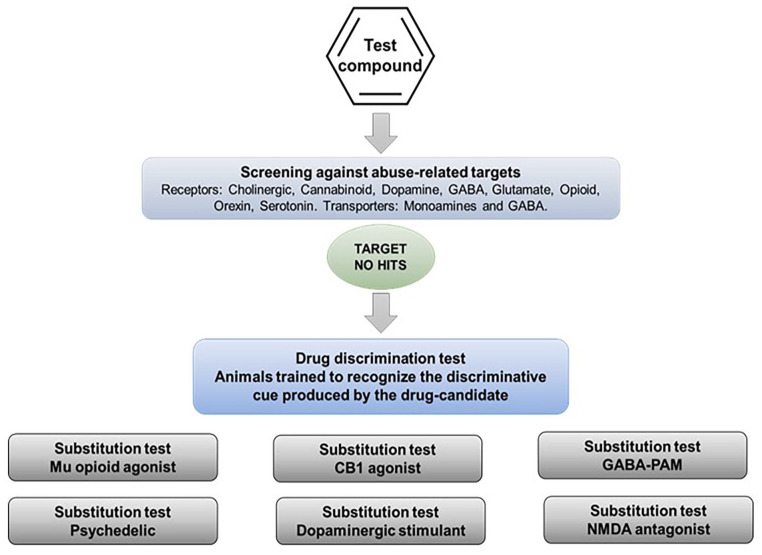
Alternative experimental design 2. Drug-discrimination test using animals that have been trained to discriminate the drug candidate from vehicle. Experimental design recommended when the therapeutic mechanism of the drug candidate is not linked with the pharmacology of known substances of abuse and screening for off-target effects yields no “hits.” Animals are trained to recognize the interoceptive cue produced by the drug candidate (positive control) and discriminate it from vehicle (negative control). Representatives of the major types of substances of abuse are tested to determine whether they substitute for the drug candidate in the model.

In a situation where the therapeutic mechanism of the drug candidate is not associated with an abuse risk and screening for off-target effects has yielded no “hits” against known drug abuse targets, regulators have asked sponsors to conduct drug-discrimination testing using the drug candidate as the discriminative cue (Figure 3). Once animals have been trained to discriminate the drug candidate from vehicle, representatives of the major types of substances of abuse would be tested to determine whether they substitute in the model. This variant is recommended on the basis that it is more cost and time efficient and saves on animal usage.

**Figure 4.**
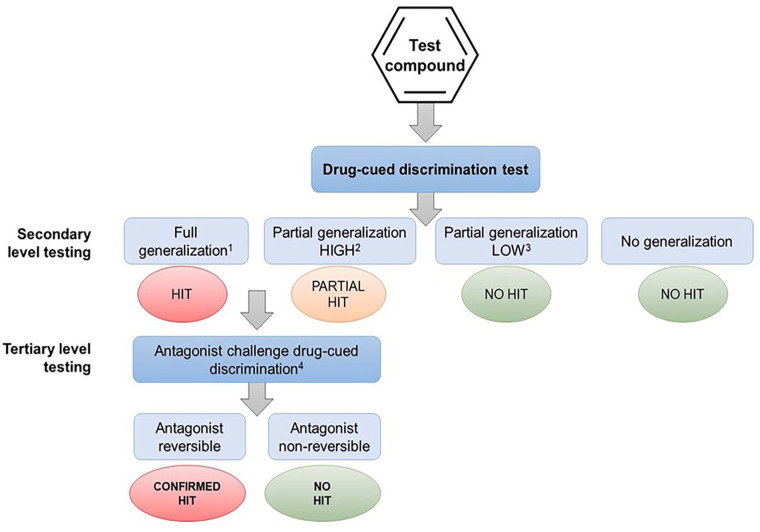
Pharmacological validation of speculative hits from drug-discrimination tests that have been obtained Alternative experimental design 1 or 2 (see text for details). ^1^Full generalization ⩾80% or ⩾75% substitution according to the model used. ^2^High-level partial generalization ⩾60% substitution. ^3^Low-level partial generalization <60% substitution. ^4^Pharmacological validation of a speculative hit that has been generated in a test where the drug candidate and any major metabolites have no affinity for the molecular target that produces the psychoactive effects of the substance of abuse used as the positive control.

This experimental design suffers from the same flaws as testing the drug candidate in series of drug-discrimination studies using a different type of substance of abuse in each one. If the drug candidate (and any major metabolites) has no affinity for known abuse-related targets, the associated substance of abuse will not generalize to the stimulus generated by the drug candidate. This point is exemplified by the work of Svedberg and colleagues (Swedberg and Raboisson, 2014; Swedberg et al., 2014) who tested a range of highly selective mGluR5 antagonists in a series of conventional drug-discrimination experiments, and also used some of these compounds as discriminative cues with substitution testing by known drugs of abuse. The results in Table 7 show identical outcomes — the mGluR5 antagonists do not substitute for the abused drugs no matter which design is used. It is important to emphasize that even if an abused drug substitutes for the drug candidate, further pharmacological characterization is necessary to define the psychoactive properties of the latter. In this situation, we recommend an additional layer of testing be performed to demonstrate that cross-substitution is pharmacologically specific (Figure 4). Thus, blocking generalization by morphine to a drug candidate’s cue by naloxone would confirm that a µ-opioid receptor mediated mechanism is responsible.

**Table 7.** Comparison of drug-discrimination results obtained using the test article as the training cue versus the use of an archetypical example of a known substance of abuse.

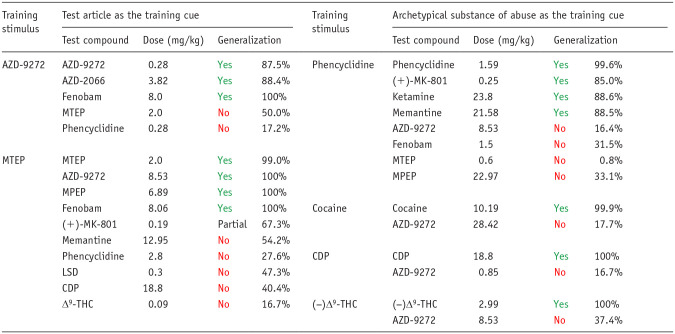

Source: Data sourced from (Swedberg and Raboisson, 2014; Swedberg et al., 2014).

Results are the mean maximum percentage generalization to the training drug cue plus the relevant dose (mg/kg). No: achieved the criterion for full generalization (>80% responding on the drug-assigned lever). Yes: the lack of cross-substitution between mGluR5 antagonists and known substances of abuse.

CDP: chlordiazepoxide; (−)Δ^9^-THC: (−)Δ^9^-tetrahydrocannabinol; LSD: lysergic acid diethylamide; MPEP: 2-methyl-6-(phenylethynyl)pyridine; MTEP: 3-[(2-methyl-1,3-thiazol-4-yl)ethynyl]pyridine.

Other issues arise; for example, what dose of the drug candidate should be used to train the animals? The CDER/FDA, (2017) guidance recommends that abuse tests should be conducted using a dose of the drug candidate that yields plasma levels 2–3× higher than the Cmax in humans at the highest therapeutic dose. To comply with this aim, we would recommend the dose of the drug candidate used to train the animals be selected to produce exposure no greater than 5× the clinical Cmax. If the training dose is set higher, the resulting discriminative cue will be relevant to toxicology not to safety pharmacology, which is in contravention of the CDER/FDA, (2017) guidelines. Moreover, because drug discrimination is an operant task, the selected dose should not markedly suppress the number of lever-presses made by the rats in training sessions. Based on our experience, >50% reduction of lever pressing for food rewards compared with vehicle or <100 lever-presses/session would be the limit for reliable responding. If the drug candidate does not elicit a robust discriminative cue, no substitution testing with known drugs of abuse is feasible and the experiment can be terminated. How should this outcome be interpreted? It is a given that CNS drugs are abused because they produce alterations in mood, sensory and/or perceptual distortions, and changes in behavioral state (activation or sedation), motivation, or disinhibition that are experienced as rewarding in humans. Those psychoactive effects, or some of them at least, underpin the drug’s ability to produce a discriminative cue in animals (Table 1). Therefore, we would propose that if a drug candidate does not elicit a discriminative cue that rats or monkeys can differentiate from vehicle, it is not a “failed study” because the outcome demonstrates that the drug candidate is devoid of psychoactive properties that could lead to abuse, c.f. no separation from placebo on key abuse-related scales in a human abuse liability trial. Based on the analysis we performed, the probability of a known substance of abuse generalizing to the discriminative cue produced by a drug candidate is vanishingly small. However, the observation that a novel drug candidate produces a discriminative stimulus demonstrates it produces recognizable psychoactive effects. However, it does not imply the drug candidate has abuse potential because discriminative stimuli can also be produced by drugs that pose no abuse risk, for example, antipsychotics (Goudie and Smith, 1999; Porter and Prus, 2009) and SSRIs and other antidepressants (Dekeyne and Millan, 2003; Wolff and Leander, 1999). If this finding is accompanied either by positive reinforcing effects in the intravenous self-administration study and/or by the occurrence of high-level abuse-related adverse events in clinical studies (a comprehensive list is available from the FDA), collectively, the evidence predicts a potential abuse risk. If the ability to produce a discriminative cue is not accompanied by other abuse potential signals, this finding would not in our view be sufficient to warrant investigating the drug candidate in a human abuse potential study.

Overall, the evidence supports the conclusion that investigating whether the drug candidate produces a discriminative cue provides insights into its potential for human abuse, especially when the result is considered in combination with information from intravenous self-administration and clinical studies. In contrast, testing representative compounds from the major classes of abused drugs to determine whether they generalize to the drug candidate’s discriminative cue will not enhance the quality of the data; rather, it could generate unreliable similarities that will mislead and confuse the abuse potential assessment.

## Conclusions and implications for the use of drug discrimination to evaluate CNS drug candidates with novel pharmacological mechanisms

We acknowledge that drug candidates with novel pharmacological mechanisms are especially problematic when it comes to evaluating whether their psychoactive properties pose a significant risk of abuse. Designing drug-discrimination protocols with no points of reference to the pharmacological mechanisms of known substances of abuse is a significant challenge. We have conducted a comprehensive analysis of the pharmacological foundations of the drug-discrimination test and have complemented it with results from experiments performed on a comprehensive panel of CNS compounds. When these findings are applied to alternative designs for drug-discrimination experiments, they support the following conclusions:

*Standard experimental design*: The standard model where animals are trained to discriminate a specific abused substance from vehicle is underpinned by decades of experimental research and has the highest degree of translational validity. Results for a drug candidate with a pharmacological mechanism that is identical or overlaps substantially with a known substance of abuse will generate robust and reliable data. If the drug candidate has an off-target interaction with an abuse-related target, the chances of full or high-level partial generalization will be greatly reduced. The published literature and our experience have taught that selecting a substance of abuse for use as a positive control based on similarities between the neuro-behavioral profiles of the drug candidate and the drug of abuse invariably produces low-level partial generalization at most. Based on the pharmacological principles underpinning the drug-discrimination test, this outcome is the correct result. On the other hand, it also raises the question of whether drug discrimination is the appropriate tool for evaluating compounds with this pharmacological profile.

*Alternative experimental design 1*: Testing drug candidates in a series of separate experiments each using a different substance of abuse as the discriminative cue has no pharmacological basis. The paradigm is only translationally valid when the drug candidate or a major metabolite shares the same pharmacology as the abused substance. In this paradigm, substitution testing against other classes of abused drugs has no predictive validity unless substitution by the drug candidate is mediated by the same pharmacological mechanism as the selected substance of abuse, for example, blocking a drug candidate generalizing to DOI’s cue by pretreatment with MDL100,907. The evidence that we have presented unequivocally shows that behavioral similarity, for example, sedation or stimulation, will not yield translationally meaningful substitution.

*Alternative experimental design 2*: Employing the drug candidate as the training cue and performing substitution testing with representative compounds from the major pharmacological classes of abused drugs is the converse of the paradigm above. This experimental design has been used in academic research, and the evidence reveals similar uncertainties as a predictor of abuse potential. Hence, decisions based on full or partial substitution without thorough pharmacological characterization (see Figure 4) have no scientific foundation or translational validity. That said, investigating whether a drug candidate generates a robust interoceptive cue at pharmacologically relevant doses will offer valuable insights into its potential for abuse. If a drug candidate with a novel pharmacological mechanism does not elicit a discriminative cue that animals can discriminate from vehicle, it does not equate to a “failed study”; rather, the outcome indicates that the drug candidate is devoid of psychoactive properties that could lead to abuse. Conversely, demonstrating that a drug candidate elicits a discriminative stimulus does not automatically imply abuse potential because stimulus cues are produced by many drugs that pose no abuse risk. In this situation, further investigation is required.

A number of serious consequences follow from using inappropriate drug-discrimination models. Unreliable models generate translationally invalid results that lead to false conclusions about the psychoactive properties and potential for abuse of CNS drug candidates. These model variants are not mentioned in the (CDER/FDA, 2017) guidance, and their proposed use is contradicted by the statements in the guidance describing the pharmacological specificity of the drug-discrimination test. The use of translationally invalid drug-discrimination models also impinges on the sensitive topic of the ethical use of animals in medical research. While research on animals to assess the safety risks to patients and the general public of novel drugs can be defended, there is no justification for their use to generate unreliable data of dubious predictive value.

## Recommendations for drug-discrimination testing

**Figure 5.**
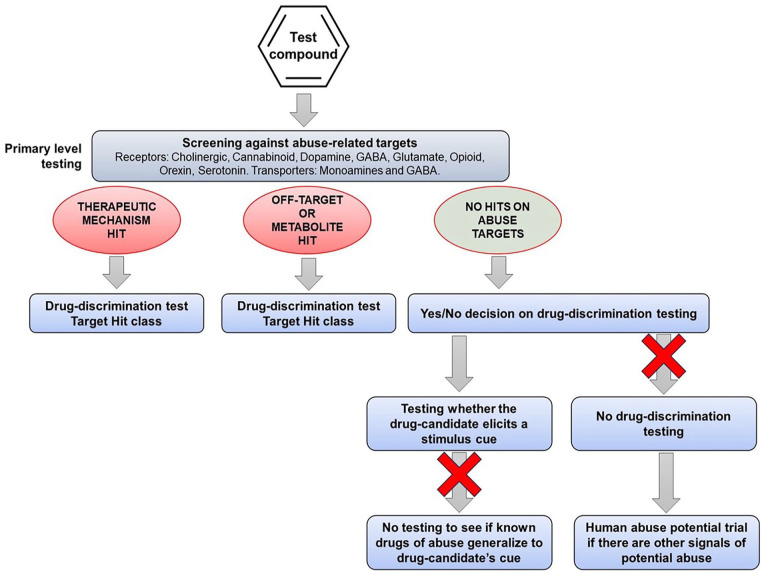
Recommendations for decisions on drug-discrimination tests based on pharmacological profiling of the drug candidate.

Based on a comprehensive review of the literature, our recommendations for conducting drug-discrimination testing to investigate the psychoactive properties of CNS drug candidates including those acting on novel CNS targets are shown in Figure 5.

If the therapeutic mechanism of drug candidate is identical or related to the actions of a known substance of abuse or a pharmacological class of substances of abuse, the standard drug-discrimination model should provide results that will be robust and reliable. When translating the findings to humans, it is important to take into account physiological differences between species. Rats do not have an emetic reflex, and this reflex is weak in primates; hence, a compound that substitutes for an abused drug that will induce nausea and emesis in humans is likely to be generating an exaggerated abuse signal in animals.

If screening of the drug candidate produces a “hit” against an abuse-related target that represents a potential “off-target” interaction, the standard drug-discrimination model is appropriate. However, based on our findings, the likelihood of observing full or high-level partial substitution will be markedly reduced. Based on our review of the evidence, this result is almost certainly translationally valid.

If screening against a comprehensive panel of abuse-related targets generates no “hits,” the evidence supports the view that speculative testing of the drug candidate in one or more standard drug-discrimination tests is unlikely to produce any reliable data. If high-level partial or full generalization occurs, it will require pharmacological validation before translating the significance of the finding to humans. Even when a positive control is selected based on similarity between the neuro-behavioral and psychoactive effects of the drug candidate and the substance of abuse, the probability of observing full or high-level partial substitution is very low. Hence, the scientific rationale for conducting drug-discrimination testing is debatable.

If the drug candidate shows positive reinforcing effects in the intravenous self-administration test or produces CNS adverse events indicative of abuse in humans and it is in development in the USA, our recommendation would be to skip the drug-discrimination test in favor of obtaining more meaningful information from a trial in drug-experienced human volunteers. Although many territories do not include a human abuse trial in their abuse/dependence testing programs, they will accept this information as part of a regulatory submission. The proposal that data from drug-discrimination test aid the design of the human abuse trial is greatly exaggerated: first, unreliable data from an invalid drug-discrimination experiment are not a sound foundation for designing a clinical trial, and second, C-I abused drugs often used in nonclinical drug-discrimination tests are not considered by regulatory authorities to be appropriate for use in human abuse trials to evaluate drug candidates for clinical approval.

The drug-discrimination test remains a central plank of the abuse potential evaluation of novel CNS drugs, but its pharmacological specificity restricts its usefulness when dealing with CNS drug candidates that have pharmacological mechanisms that have not previously been explored in humans and are unrelated to those of known substances of abuse. We reviewed the model variants that have been recommended for abuse evaluations and critically analyzed their scientific rationale and translational validity. Having determined that two of these model variants do not meet these acceptance criteria, we have made constructive proposals on when and how to use the drug-discrimination test to evaluate CNS drugs for medical use. Our view is the drug discrimination still has an important role in safety pharmacology, but the model and its application need to be adapted to meet the challenges of CNS drugs with novel mechanisms. Failure to adapt its use will inevitably lead to a downgrading of its predictive value and the valuable contribution that it makes to abuse potential assessments when used appropriately.
